# Early and Multimodal Care for Patients with Lung Cancer: Findings from the ACCEPT Study

**DOI:** 10.3390/cancers18193081

**Published:** 2026-09-22

**Authors:** Christian Grah, Shiao Li Oei, Felix Kindler, Alexander Krabbe, Hannah Wüstefeld, Marcus Reif, Anja Thronicke, Steven Ngandeu Schepanski, Georg Seifert, Friedemann Schad

**Affiliations:** 1 Lung Cancer Center, Hospital Gemeinschaftskrankenhaus Havelhöhe, Kladower Damm 221, 14089 Berlin, Germany; hannah.wuestefeld@havelhoehe.de; 2 Research Institute Havelhöhe, Hospital Gemeinschaftskrankenhaus Havelhöhe, Kladower Damm 221, 14089 Berlin, Germany; shiaoli.oei@havelhoehe.de (S.L.O.); felix.kindler@gmx.net (F.K.); alexander.krabbe@havelhoehe.de (A.K.); anja.thronicke@havelhoehe.de (A.T.); fschad@havelhoehe.de (F.S.); 3 Charité Competence Center for Traditional and Integrative Medicine (CCCTIM), Charité—Universitätsmedizin Berlin, 10117 Berlin, Germany; steven.ngandeu-schepanski@charite.de (S.N.S.); georg.seifert@charite.de (G.S.); 4 Gesellschaft für Klinische Forschung e.V., 10623 Berlin, Germany; marcus.reif@gkf-berlin.de; 5 Department of Pediatrics, Division of Oncology and Hematology, Charité—Universitätsmedizin Berlin, 13353 Berlin, Germany; 6 Interdisciplinary Oncology and Palliative Care Center, Hospital Gemeinschaftskrankenhaus Havelhöhe, 14089 Berlin, Germany

**Keywords:** early supportive care, fatigue, lung cancer, psychological distress, quality of life

## Abstract

**Objective**: Treating lung cancer with a combination of targeted cancer therapy, supportive, and psychosocial treatments is important in modern oncology. The ACCEPT study investigated the efficacy of a 12-week early multimodal supportive care program on quality of life. **Methods**: ACCEPT was a single-center, non-randomized, prospective, open-label trial, with a pragmatic real-world design guided by patient choice. Patients with newly diagnosed, stage II-IV lung cancer participated in the outpatient ACCEPT program (psycho-oncology, health education, and training-based interventions alongside standard care). Longitudinal patient-reported outcome measures (PROMs) were collected. The primary outcome was the change in quality of life at 12 weeks measured by the Trial Outcome Index (TOI). Secondary outcomes included further exploratory PROM changes and overall survival. **Results**: Between April 2018 and August 2021, 214 lung cancer patients (median age 67, 53% male, 57% stage IV) were enrolled, with 116 patients evaluable for longitudinal PROM changes at 12 weeks. Participation in the multimodal ACCEPT program was associated with up to 17% improvement in the TOI, psychological distress, and sleep quality (*p* < 0.0001). Multivariate analyses revealed that greater session attendance was significantly and dose-dependently associated with improvements in the TOI as well as reductions in depression and anxiety. Higher engagement in multimodal sessions was also linked to reduced fatigue. Furthermore, exploratory signals of improved overall survival were observed among the 40 patients (18.7%) who fully completed the ACCEPT program. **Conclusions**: This pragmatic, patient-centered, multimodal supportive care program for lung cancer patients was associated with dose-dependent improvements in quality of life and psychological distress. While exploratory signals of improved survival were observed, multicenter randomized controlled trials are required to confirm these findings and firmly establish the role of such supportive care programs in routine oncology.

## Simple Summary

In modern oncology, treating lung cancer with a combination of medical, supportive, and psychosocial treatments is important. The ACCEPT study examined how a 12-week multimodal early cancer care training program influences quality of life. In this study, directed by patient choice, participation in the ACCEPT program over twelve weeks was associated with an improvement in quality of life. Engagement in multimodal ACCEPT sessions was associated with improved quality of life, reduced depression and symptom burden, and signals of extended survival. The findings of this study indicate that this multimodal training ACCEPT program may be an effective intervention for supporting quality of life and reducing multiple burdens in lung cancer patients.

## 1. Introduction

Despite significant therapeutic advances in cancer treatment, lung cancer (LC) remains the leading cause of cancer-related mortality worldwide [1]. Advanced LC is a debilitating malignancy characterized by an exceptionally high symptom burden, profound psychological distress, and a severely compromised quality of life (QoL) that often surpasses other oncological subtypes [2]. To optimize clinical prognosis and mitigate this burden in contemporary oncology, both standard of care (SOC) and supportive care regimens must be strategically aligned within a patient-centered framework.

A compelling biological rationale for this integration is provided by psychoneuroimmunology, where accumulating evidence underscores the capacity of psychosocial interventions to alter immune system dynamics. Broad meta-analytic data confirm that these non-pharmacological approaches significantly mitigate pro-inflammatory signaling pathways while fortifying host protective immunity [3,4]. These distinct immunomodulatory effects—particularly evident in the reduction in systemic inflammatory markers following cognitive behavioral interventions—demonstrate a sustained capacity to optimize the biological microenvironment for several months [3,4].

Translating these biobehavioral dynamics into clinical practice, the seminal work by Temel et al. (2010) [5], alongside subsequent large-scale randomized controlled trials globally [6,7,8,9], provides definitive empirical validation for the early integration of supportive interventions [10]. In patients with advanced malignancies, such concurrent care not only yields significant improvements in psychological well-being but, crucially, confers a measurable survival advantage over SOC alone. In addition to early supportive care, structured advance care planning utilizing patient-centered communication and decision aids further enhances QoL [11].

Consequently, major oncology guidelines now mandate that patients receive comprehensive psycho-oncological and non-pharmacological supportive care concurrently with standard cancer therapies [12]. However, translating these established benefits specifically to the broader LC landscape reveals critical evidence gaps. A recent systematic review evaluating the quality of LC care identified 30 studies assessing early palliative care [13]. Although this analysis indicated a potential improvement in overall survival, the underlying study data were characterized by low methodological quality and high heterogeneity, thereby limiting the feasibility of a robust meta-analysis for QoL outcomes and underscoring the need for more rigorous, standardized investigation. Reflecting this lack of standardization, the optimal configuration of early supportive components remains poorly defined despite broad consensus on its integration.

Although the key elements of integrated palliative care for chronic respiratory conditions have recently been synthesized [14], determining the precise combination of modules for early supportive oncology necessitates a meticulous design that maximizes therapeutic benefit while avoiding patient overburden. To address this paradigm, the ACCEPT program was developed, incorporating a multifaceted range of interventions structured into three core modules. While previous work has demonstrated the feasibility of delivering this program alongside SOC [15], its clinical application requires a departure from traditional, rigid frameworks. Historically, early palliative care for advanced malignancies has been initiated within a fixed timeline, typically within eight weeks of diagnosis [5]. However, contemporary evidence suggests that early interventions should be guided by individual symptom trajectories and patient needs rather than static, prognosis-based chronological criteria. Furthermore, supportive care models must continuously adapt both to the rapid evolution of oncological therapeutics and to the shifting needs of patients as the disease progresses [14]. Driven by the hypothesis that patient empowerment and psychoneuroimmunological outcomes are profoundly enhanced by the self-selected, individualized initiation of supportive care modules, the ACCEPT study adopted a pragmatic preference-based design rather than a randomized allocation. Consequently, this study aimed to assess whether a patient-preference-based program modulates psychoneuroimmunology pathways and yields measurable improvement in patient QoL.

## 2. Materials and Methods

The Additive Integrative Medicine Cancer Concept of Early Supportive or Palliative Lung Cancer Treatment (ACCEPT) study was an investigator-initiated, prospective, longitudinal, single-center, open-label trial conducted at the German Cancer Society-accredited Lung Cancer Center at the hospital Gemeinschaftskrankenhaus Havelhöhe in Berlin, Germany. The study protocol was approved by the Ethics Committee of the hospital Charité—Universitätsmedizin Berlin (application number EA1/231/16) on 13 October 2016 and was most recently amended on 2 September 2020.

The study population consisted of patients with a primary, histologically proven diagnosis of LC with Union for International Cancer Control (UICC) stage II–IV, diagnosed within the previous six weeks, with an Eastern Cooperative Oncology Group (ECOG) performance status of ≤2. Patients were informed of the details of the ACCEPT study and were invited and encouraged to participate in the program. All participants provided written informed consent. Participants were not compensated. Using a pragmatic, retrospective approach, patients were allocated into three groups based on their level of participation and consent. The SOC control group comprised patients unable or unwilling to engage in the ACCEPT interventions but capable of completing PROMs. Importantly, patients who did not participate in the ACCEPT program maintained full access to other supportive-care or psycho-oncological services as part of routine clinical care. The multimodal treatment group consisted of patients who consented to and participated in the full ACCEPT program. Patients who consented to the program but did not engage with module 3 were assigned to the bimodal group.

All patients were treated in accordance with prevailing oncology guidelines, the recommendations of the multidisciplinary tumor board and patient preference. According to the ACCEPT feasibility study [15], a multimodal treatment approach was used. The ACCEPT program is structured as a 12-week, multimodal, preference-based intervention designed to enhance patient autonomy, clinical coping mechanisms, and QoL, alongside SOC. The concurrent delivery framework of the three core modules is schematically illustrated in Figure 1.

Module 1 consisted of 12 weekly, 120 min health education sessions delivered in a rotating sequence. These sessions addressed a broad range of medical and psychological topics, including nutrition, self-treatment strategies, breathing exercises, stress management, resource optimization, and relaxation techniques. Conducted in a group setting, the sessions actively encouraged peer discussion and interactive resource-management exercises. The health education program was coordinated by a dedicated nursing team and supplemented by invited clinical experts.

Module 2 comprised psycho-oncological counseling tailored for individuals or families, consisting of three 45 min sessions scheduled across the 12-week intervention period. Additional consultations were available upon request or under exceptional circumstances, and partners were invited to participate. Conducted by a psycho-oncologist, these open discussions followed a structured curriculum designed to address seven key thematic areas:

1. Anxiety and Avoidance Behavior: Managing fears and avoidance patterns;
2. Substance Use and Addiction: Addressing tobacco dependence and other addictive behaviors;
3. Social Stress and Support: Evaluating social networks and developing utilization skills;
4. Individual Illness Appraisal: Exploring personal meaning, future perspectives, and the impact on the family/social network;
5. End-of-Life Discussions: Navigating mortality, coping strategies, and advance care planning;
6. Resource Mobilization: Identifying personal strengths and sources of resilience;
7. Crisis Management: Developing specific coping skills and crisis-intervention strategies.

Furthermore, the first and final sessions incorporated the Pictorial Representation of Illness and Self-Measure (PRISM) tool [16] to visualize the patient’s perceived proximity to or distance from their illness. This visualization was integrated into the counseling sessions based on patient preference. Therapists documented highly relevant discussion topics to guide follow-up during subsequent consultations.

Module 3 features an integrated, home-based creative art therapies (ARTs) program developed by an interdisciplinary panel of oncologists and specialized creative therapists [15]. Aimed at fostering patient empowerment and self-confidence, the module focuses on inner spiritual strengthening and self-awareness rather than physical exertion. Between weeks 6 and 12, patients attend four 45 min supervised training sessions at the lung cancer center to prepare them for independent, daily 10 min home practice over a six-week period. A written handout guided home adherence, which patients recorded in a structured diary. Patients selected one of three anthroposophic integrative medicine modalities adapted for lung cancer care: (1) eurythmy therapy, (2) therapeutic speech and breathing training, or (3) singing and musical breathing therapy. Specifically, eurythmy therapy was applied as an integrative movement modality utilizing specific vocal and musical gestures to dynamically regulate breathing patterns, restore physiological rhythms, and optimize respiratory economy without physical exertion. Completed diaries were collected by the study center at week 12 for compliance analysis.

The study utilizes the Functional Assessment of Cancer Therapy-Lung (FACT-L) scale to measure the primary outcome of 12-week changes in the Trial Outcome Index (TOI) [17]. In accordance with landmark trials in early supportive care (e.g., Temel et al. [5]), the TOI was selected as the primary endpoint because physical and functional capacities represent the most immediate clinical burden in lung cancer patients. Broader psychosocial and spiritual domains were evaluated using secondary endpoints: the Hospital Anxiety and Depression Scale (HADS) [18], Pittsburgh Sleep Quality Index (PSQI) [19], and Cancer Fatigue Scale (CFS-D) [20]. This battery of PROMs (Table 1) was administered at baseline (T1), 45 days (T2), and 90 days (T3), followed by long-term follow-ups at 6, 12 and 24 months, spanning a total period of up to three years.

The study was powered at 80% to detect a clinically relevant difference in the primary outcome between the SOC and multimodal treatment groups. As the dropout rate exceeded expectations, the steering committee extended enrollment until August 2021 to achieve a target sample of at least 200 patients. Demographic and diagnostic variables were collected, and all characteristics and treatments were summarized descriptively. No imputation method was performed for missing or lost-to-follow-up data. The baseline characteristics were compared between treatment groups using the chi-squared test. For the analyses of PROMs, the starting date was the recorded date of study entry (index date). Two-sided *p*-values of 0.05 or less were considered statistically significant. Changes in PROMs were evaluated using multivariate linear regression analyses. A two-step approach was utilized for variable selection: factors demonstrating a relevant association in the initial univariate analyses were subsequently included in the final multivariate models. Separate regression analyses were conducted for each PROM score, with the resulting beta estimates reported. Given the exploratory nature of these analyses, no adjustment for multiple testing was performed; accordingly, *p*-values are reported as nominal and should be interpreted descriptively rather than as confirmatory evidence. Prior to statistical inference, standard model assumptions were assessed and found to be adequately met.

To account for potential differences in baseline characteristics due to the non-randomized allocation of participants, inverse probability of treatment weighting (IPTW) was applied using multinomial propensity scores. Preliminary analyses indicated that while key predictors of overall survival—such as tumor stage—strongly influenced baseline PROMs, they did not substantially impact subsequent PROM changes. In fact, baseline PROM scores, sex, and ECOG performance status emerged as the most relevant predictors of PROM trajectories. Consequently, these variables were selected as the primary covariates for weight generation. Weighted linear regression models were then used to estimate group differences in PROM changes (ΔTOI and ΔHADS). We assessed the balance of covariates before and after weighting using standardized mean differences (SMDs), considering values of less than 0.1 to be indicative of adequate balance.

For survival analyses, time zero was defined as the date of the primary histological diagnosis of LC. Overall survival was estimated using the Kaplan–Meier method. To evaluate the independent effects of the intervention while controlling for potential confounding factors, multivariable Cox proportional hazards models were subsequently fitted. This regression approach is specifically designed to model time-to-event data, allowing for the simultaneous assessment of multiple covariates on the hazard rate, while appropriately accounting for right-censored data. In our models, data were censored at 60 months. Before conducting the multivariate Cox proportional hazards ratio modeling, rigorous verification analyses were performed to ensure that the underlying proportional hazards assumptions were statistically satisfied. All statistical analyses were performed using Excel 2010 (Microsoft, Redmond, WA, USA) or R (version 4.2.1, R core team, Vienna, Austria). The generation of figures and survival curves was performed in R (version 4.2.1) [21] using the survival (version 3.4-0), ‘survminer’ (version 0.4.9) and ‘ggfortify’ (version 0.4.14) packages.

## 3. Results

### 3.1. Patient’s Characteristics

A total of 1200 patients with lung cancer were treated at the GKH Lung Cancer Center between 2018 and 2021. Of these, 737 patients with UICC stage II–IV were screened and 234 patients provided written informed consent and met preliminary eligibility criteria. Twenty patients were subsequently excluded due to withdrawal of consent or non-eligible histology. This left 214 patients who initiated standard oncology treatment and completed baseline PROM assessments (Figure 2). During follow-up, the number of patients completing PROM assessments decreased over time: 116 patients returned PROMs at 12 weeks and were included in the PROM-change analysis, followed by 81 patients at 6 months, and 57 patients at 12 months. All dropouts across these time points are detailed in the flowchart in Figure 2. Overall survival analyses comprised all 214 patients.

All patients received care in accordance with current oncology guidelines and multidisciplinary tumor board recommendations, with treatment decisions reflecting clinical and oncological characteristics, disease courses, and patient preferences. Group allocation followed pragmatic criteria. Patients who declined participation in the ACCEPT program but agreed to complete the questionnaires were assigned to the SOC (control) group. Patients who participated in all three modules were assigned to the multimodal treatment group, while those who enrolled in the program but declined module 3 were classified as the bimodal group. Key baseline characteristics for the three groups are shown in Table 2.

The following changes were made to the study protocol: Between 2020 and 2021, delays in recruiting study participants and implementing the ACCEPT modules were caused by the SARS-CoV-2 pandemic, necessitating adjustments to the organization of the intervention. Group interventions were either canceled or, in a few cases, replaced by individual consultations. However, as far as possible, the oncology health education program was maintained, and appointments were conducted in compliance with social distancing rules and with face masks.

Between March 2020 and February 2021, during the peak of the pandemic, only 63 patients could be recruited for the ACCEPT study. Of these patients, only five (8%) were able to participate in all three modules, compared to 18.7% of the entire cohort who engaged with the complete multimodal ACCEPT program.

Overall, the ACCEPT program was applied less frequently during the pandemic compared to the periods before and after. A total of 98 patients did not complete the 12-week PROM assessment (Figure 2). Of these non-responders, 18 had died, and 80 were unable or unwilling to participate owing to poor condition, limited time, organizational constraints, pandemic-related difficulties, or lack of interest.

A total of 116 patients (54.2%) returned the 12-week PROMs, and their data sets were included in the analysis. A significantly higher proportion of women than men completed the PROMs (*p* = 0.005). Apart from sex, no significant differences were observed between responders and non-responders (Table 3).

While the baseline characteristics for the three ACCEPT groups are shown in Table 2, additional details are provided in Table 4.

### 3.2. Assessing Longitudinal PROM Changes

The ACCEPT program comprises twelve sessions of module 1, three sessions of module 2, and four sessions of module 3. The number of sessions could be adapted according to individual patient preferences and is summarized in the lower part of Table 3 for the entire cohort and in the upper part of Table 5 for the PROMs-responding cohort. The 12-week PROM analysis included 116 patients (SOC, n = 43; bimodal, n = 38; multimodal, n = 35). Mean baseline scores and 12-week changes are shown in Table 5.

No meaningful changes were observed in the SOC control group, where scores appeared to remain stable. In contrast, the multimodal group showed substantial and statistically significant improvements across all analyzed PROMs (8% to 17%; see Table 5). The bimodal group also demonstrated significant, albeit less pronounced, improvements (6% to 11%; see Table 5). Baseline group differences in demographic characteristics (Table 2) were statistically controlled for in subsequent multivariable models. These observed PROM improvements showed a similar pattern overall when comparing the unadjusted findings in Table 5 with the propensity score-weighted estimates in Table 6, suggesting the findings are not heavily skewed by baseline differences. Specifically, within the weighted models, participation in the multimodal program was associated with significant improvements in both physical function and well-being (TOI: +11.52; *p* = 0.002) and psychological distress (HADS: −6.18; *p* < 0.001). For the bimodal group, a significant association was found only for the reduction in psychological distress (ΔHADS = −3.86; *p* = 0.016), whereas the corresponding change in physical function and well-being did not reach statistical significance (ΔTOI = +3.83; *p* = 0.298; see Table 6).

Sustained improvements appeared to be maintained during the follow-up period. At six months, participation in the multimodal program was associated with estimated improvements relative to baseline of 11% in physical function and well-being (TOI; *p* = 0.004), 12% in psychological distress (HADS; *p* < 0.0001), and 9% in sleep quality (PSQI; *p* = 0.006). At twelve months, these favorable trends remained statistically significant: 10% in physical function and well-being (TOI; *p* = 0.04), 9% in psychological distress (HADS; *p* = 0.005), and 6% in sleep quality (PSQI; *p* = 0.017); see Table 7.

A series of multivariate analyses was performed to identify potential dose–response associations between PROM changes and the number of sessions attended in each program module. The upper portion of Table 8 presents representative models assessing the potential dose–response relationships between individual modules and specific outcomes (HADS with Module 1, FACT with Module 2, and TOI with Module 3). Within these modules, attendance in Module 1 appeared to be associated with an estimated 0.8% improvement in the psychological distress total score (HADS) per attended session. Similarly, each Module 2 session was associated with an estimated 3.4% improvement in the lung cancer-specific quality of life total score (FACT-Lung Total), and each Module 3 session corresponded to a predicted 3.1% improvement in the physical function and well-being (TOI). The association factors regarding the number of module sessions attended are summarized across all PROM changes in the lower portion of Table 8.

Across the analyzed PROMs, session attendance showed indications of both clinically meaningful and statistically significant improvements, although these trends varied, appearing more defined during Modules 2 and 3 (yielding improvements of approximately 3% to 4% per session).

Additionally, within the scope of Module 3, each attended session was associated with an estimated 2.3% reduction in reported fatigue severity (Table 8, lower panel).

Specifically, notable associations were identified for the HADS-Anxiety subscale, which showed estimated improvements of 0.8%, 4.1%, and 3.7% per session attended for Modules 1, 2, and 3, respectively. Additionally, within the scope of Module 3, participation was associated with a 2.3% reduction in fatigue (Table 8, lower panel).

At the six-month follow-up, indications of these dose–response relationships were still present across the physical function and well-being (TOI), psychological distress (HADS), and sleep quality (PSQI) scales (Table 9). At the 12-month follow-up, each additional Module 2 session remained associated with a 4.1% improvement in the TOI score, though the statistical significance was marginal (*p* = 0.04; Table 9).

### 3.3. Evaluation of Overall Survival

By the cut-off date, death had occurred in 177 of the 214 patients. In the 5-year survival analysis, the median overall survival for the entire cohort was 16.9 months (95% CI: 13.6–21.1).

Kaplan–Meier survival curves stratified by sex, tumor stage, ECOG performance status, and the receipt of brain irradiation are displayed in Figure 3. Within this sample, male sex, advanced tumor stages, an ECOG performance status greater than 1, and prior brain irradiation each appeared to be associated with increased risk of mortality, demonstrating statistically significant differences in survival trajectories.

A comparison of baseline characteristics indicated several notable imbalances between the study groups (Table 2 and Table 4). Within our sample, patients in the multimodal group tended to be younger, more frequently female, more highly educated, and presented with a lower burden of comorbidities, less advanced tumor stages, and lower ECOG performance status scores compared to the SOC group. Patients in the bimodal group exhibited broadly similar, though less pronounced, trends, characterized primarily by higher educational attainment, fewer comorbidities, and a more favorable ECOG status relative to the SOC cohort (Table 2). Furthermore, a higher proportion of patients with targetable genetic alterations—who consequently received targeted therapy—appeared to be allocated to the multimodal group (Table 4). Concurrently, median overall survival appeared longer in the multimodal group compared to the SOC group (36.8 versus 11.7 months; Figure 4A). Multivariate Cox regression models were adjusted for age, sex, tumor stage, ECOG performance status, and the administration of brain irradiation. Following adjustment for these potential confounding variables, the multimodal group was associated with a statistically significant lower hazard of death compared to the SOC group (adjusted hazard ratio [aHR], 0.39; 95% CI, 0.25 to 0.63; *p* < 0.001). Within the framework of this model, these data suggest an estimated 61% lower hazard of mortality (Figure 4B).

Several significant differences were identified between the groups regarding molecular pathology parameters and respective oncological treatments (Table 4). Consequently, two additional variables—the epidermal growth factor receptor (EGFR) status and the administration of immunotherapy—were incorporated into subsequent multivariable Cox regression models (Figure 5).

The potential dose-dependent relationship between overall survival and the number of sessions attended for each ACCEPT module was evaluated across the entire cohort (n = 214). These adjusted Cox analyses were performed separately for each of the three modules, as well as for the cumulative number of sessions across all modules (see Figure 5A–D; session attendance details are summarized in Table 4). As illustrated in Figure 5, receiving immunotherapy showed a potential association with a lower hazard of death (estimated at >30%, *p* = 0.01). Furthermore, lower hazard trends were observed across the models for all individual modules (Figure 5A–C). Specifically, each session of Module 2 attended was associated with an adjusted hazard of death (aHR 0.77, 95% CI: 0.68–0.88, *p* < 0.001; Figure 5B). When evaluating cumulative attendance across all modules, each additional session was associated with an aHR of 0.96 (95% CI: 0.93–0.99, *p* = 0.003; Figure 5D).

## 4. Discussion

The early, patient-centered ACCEPT program for lung cancer patients appeared to be associated with trends toward dose-dependent improvements across multiple PROMs. Our preliminary data suggest a potential correlation between QoL enhancement and the number of multimodal interventions attended. While our analyzable sample size (n = 116) was lower than the targeted 200 due to typical attrition in lung cancer trials, the observed effects were sufficient to detect statistically significant trends in PROM changes, which should be considered preliminary until confirmed by larger trials. Notably, up to 17% improvements were recorded across the evaluated PROMs—including the lung cancer-specific quality of life (FACT-L), psychological distress (HADS), sleep quality (PSQI), and fatigue (CFS-D)—as well as the primary endpoint, physical function and well-being (TOI). While an increase of >5 points (>6%) on the physical function and well-being subscale (FACT-L TOI) is widely recognized as the minimal clinically important difference (MCID) required to signal a clinically meaningful change for lung cancer patients [17], the improvements observed within the ACCEPT program appeared to substantially exceed this threshold. In comparison, the landmark study by Temel et al. [5] reported a 5-point (6%) improvement in the TOI, indicating that the effect sizes observed in our cohort are descriptively two- to three-fold larger than those historical benchmarks.

This pronounced difference could potentially be attributed to the substantial evolution of oncological care since 2010, alongside the preference-based implementation of our interventions, though direct comparisons across distinct study designs should be made with caution. Furthermore, while evidence regarding effective strategies to counteract fatigue in lung cancer remains limited, our exploratory findings suggest that a multimodel framework like the ACCEPT program—including creative art therapies (ARTs)—may offer a potential approach for this cohort, though these trends require confirmation. Interestingly, the associations observed here in lung cancer patients appear to be more pronounced than those reported for similar interventions in some breast cancer cohorts [22,23,24]. This discrepancy could be speculative, in part, due to differences in the existing healthcare landscape; while breast cancer patients typically have access to an abundance of routine support services outside of clinical trials, such resources remain comparatively scarce for lung cancer patients. Consequently, our cohort may have had a higher unmet need, potentially rendering them more responsive to the interventions provided within the framework. Although systematic reviews have established the benefits of structured exercise on QoL and depression in lung cancer [25,26,27], and meta-analyses support breathing exercises [28] and multimodal physiotherapy interventions [29] for optimizing postoperative recovery, our findings suggest that a multi-component framework might offer additional, complementary support.

We studied patients with advanced or metastatic LC and observed that the ACCEPT program may be associated with potential dose-dependent improvements in PROMs. In particular, our findings suggest possible trends regarding the Psycho-oncology and ARTs modules with respect to anxiety and physical health. The most prominent suggestion of an association was observed within the anxiety subscale, with a descriptive 1% to 4% improvement associated with each attended ACCEPT session.

Our findings should be interpreted cautiously within the framework of psychoneuroimmunological models. Through modules targeting distress, sleep, and physical functioning—as reflected in the HADS, PSQI, and FACT-L TOI scores—the ACCEPT program is conceptually designed to potentially mitigate stress-induced proinflammatory pathways. However, as no immunomodulatory biomarkers were collected in this pragmatic trial, these patient-reported improvements cannot substitute for biological metrics. The fact that improvements in physical function and well-being (TOI) and psychological distress (HADS) scores exceeded standard MCID thresholds twofold points to a meaningful psychological impact of choice-driven supportive care, which might theoretically translate into reduced proinflammatory signaling. Nevertheless, without direct physiological data, any link to actual immunomodulation remains strictly hypothetical, speculative, and purely hypothesis-generating for routine oncology settings. These clinical findings merely provide a plausible rationale for future studies to incorporate objective immunological measures to evaluate the biological impact of this program.

Our observed survival rates align with published real-world data showing global improvements in lung cancer survival from 2011 to 2020 [30]. Although the observed association between higher ACCEPT participation and prolonged overall survival is promising, these results mandate a cautious interpretation. In a pragmatic setting, treatment adherence is heavily intertwined with favorable prognostic factors. Patients surviving longer or tolerating anticancer therapy better naturally had more opportunities to attend a higher number of sessions. While we adjusted for key baseline characteristics, such as sex, cancer stage, ECOG performance, brain metastases, etc., we cannot rule out residual confounding driven by unmeasured factors, such as superior baseline health status, stronger social support networks, or higher baseline physical and psychological resilience. Consequently, this survival association should be viewed as hypothesis-generating and interpreted within the limitations of preference-based clinical designs.

The heterogeneity of our cohort, spanning early and advanced disease stages, was a deliberate choice to maximize external validity and reflect real-world clinical practice. The ACCEPT program was designed as a flexible intervention, rather than a rigid protocol. Evaluating it across this broad spectrum demonstrates how a single, multimodal framework can be tailored to diverse needs—ranging from recovery in early-stage patients to symptom mitigation in advanced palliative care. This approach highlights the generalizability and practical feasibility of the program in everyday oncology settings.

Notably, the program faced a 50% non-participation rate among invited candidates, alongside elevated attrition driven by clinical worsening or pandemic-related barriers. Consequently, future translation of the ACCEPT framework into routine oncology care requires enhanced engagement strategies. In particular, the home-based exercises and breathing therapies in Module 3 present distinct feasibility challenges that warrant further investigation.

Several limitations of the present study warrant consideration. First, the non-randomized, open-label, preference-based, and monocentric design inherently increases vulnerability to selection bias, investigator bias, and residual confounding. Consequently, this limits direct comparisons with explanatory randomized controlled trials. While we adjusted for key baseline characteristics, statistical conditioning cannot fully control for unmeasured confounding. Second, the study cohort exhibited high clinical heterogeneity, which was further compounded by environmental disruptions during the SARS-CoV-2 pandemic that impacted systematic data collection. Additionally, potential enrollment bias exists, as only 30% of eligible patients were successfully recruited. Because reasons for non-participation were not systematically documented, the final cohort may be skewed toward highly motivated individuals or those with specific clinical baselines. Third, patient engagement—specifically the number of ACCEPT modules attended—is highly endogenous. Participation is likely confounded by baseline factors (e.g., age, sex, disease severity) and unmeasured traits like intrinsic motivation, social support, or resilience, posing a risk of healthy-adherer bias. Although multivariate models adjusted for baseline ECOG status, residual confounding may persist. Consequently, we cannot fully disentangle whether the observed clinical benefits and prolonged overall survival are driven by program adherence or by underlying prognostic characteristics, such as the higher proportion of EGFR mutations and targeted therapy use in the multimodal cohort. While this limits definitive causal conclusions, the findings still offer valuable real-world insights. Fourth, a major challenge is the substantial attrition rate in longitudinal PROMs, driven by questionnaire fatigue and a sharp compliance decline in the SOC group. Due to this unevenly distributed missingness, statistical imputation was avoided. In advanced lung cancer trials, missing data are frequently non-random and heavily driven by clinical deterioration or death. As a result, patients with worsening health are systematically underrepresented at later time points. This healthy-responder or survival bias may overestimate the program’s benefit, as follow-up data predominantly reflect patients well enough to complete assessments. Fifth, due to methodological limitations, these findings must be interpreted as exploratory rather than confirmatory, and no strictly causal dose–response relationship can be stated; future randomized trials with fixed treatment doses are necessary to establish causality. Readers should, therefore, interpret the magnitude of the PROM improvements with caution within the context of this inevitable, real-world attrition.

These methodological constraints are counterbalanced by several key strengths. First, the ACCEPT study offers ecologically valid insights into preference-based, real-world supportive care within a guideline-compliant German lung cancer center. Minimal exclusion criteria ensured broad inclusivity, capturing a diverse patient population routinely excluded from strict clinical trials. Second, the inherent risk of confounding from non-randomized allocation was rigorously addressed. By employing multivariable regression, propensity score weighting, and multivariable Cox regression analyses, we systematically controlled for baseline imbalances, thereby enhancing the internal validity of our observations. Third, the study provides an initial foundation for economic viability. The net cost of the 12-week ACCEPT program was estimated at approximately €2000 per participant in addition to SOC. While future health economic evaluations are essential to confirm whether these expenditures translate into long-term savings or reduced healthcare utilization, this cost framework supports the feasibility of future implementation. Consequently, these findings offer valuable real-world evidence and provide a crucial foundation for designing future multi-center confirmatory trials.

## 5. Conclusions

The implementation of the patient-centered ACCEPT program appeared feasible within a certified lung cancer center, with data suggesting encouraging trends in quality of life, distress, sleep quality, and symptom burden—even among patients with partial compliance. Notably, cumulative module attendance was tentatively linked to favorable survival signals. However, the program faced a substantial attrition rate, and patient willingness to engage appeared to correlate with sex, tumor stage, baseline performance status, and educational level. The varying engagement reflects the intensive nature of the program for lung cancer patients with high symptom burdens. These patterns suggest that a rigid, 12-week curriculum is overly demanding. For real-world clinical implementation, a more flexible, modular approach is required, allowing patients to adapt their participation based on their immediate capacities and fluctuating needs. In order to effectively integrate the significant benefits of multimodal therapy into lung cancer care, future prospective, multicenter studies must establish a standardized framework. Only with such evidence can clinical guidelines develop targeted strategies and provide healthcare professionals with the foundation needed to offer therapy options and comprehensively implement multimodal therapy at treatment centers and across all oncology settings.

## Figures and Tables

**Figure 1:**
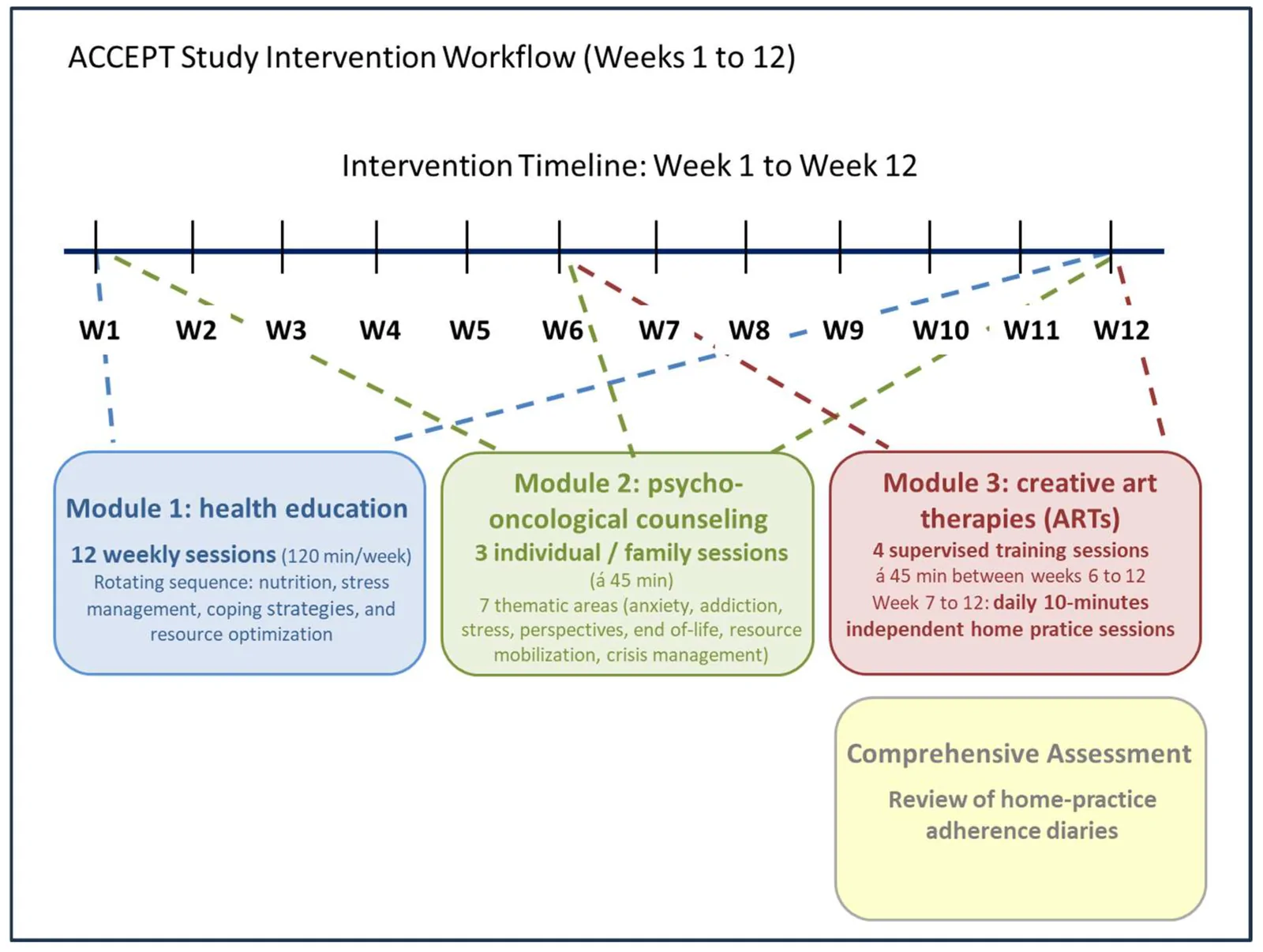
Overview of the 12-week multimodal, preference-based ACCEPT intervention program. The schematic illustrates the simultaneous delivery of Module 1 (Health Education), Module 2 (Individual/Family Psycho-oncological Counseling), and Module 3 (Creative Art Therapies (ARTs). ACCEPT: Additive Integrative Medicine Cancer Concept of Early Supportive or Palliative Lung Cancer Treatment.

**Figure 2:**
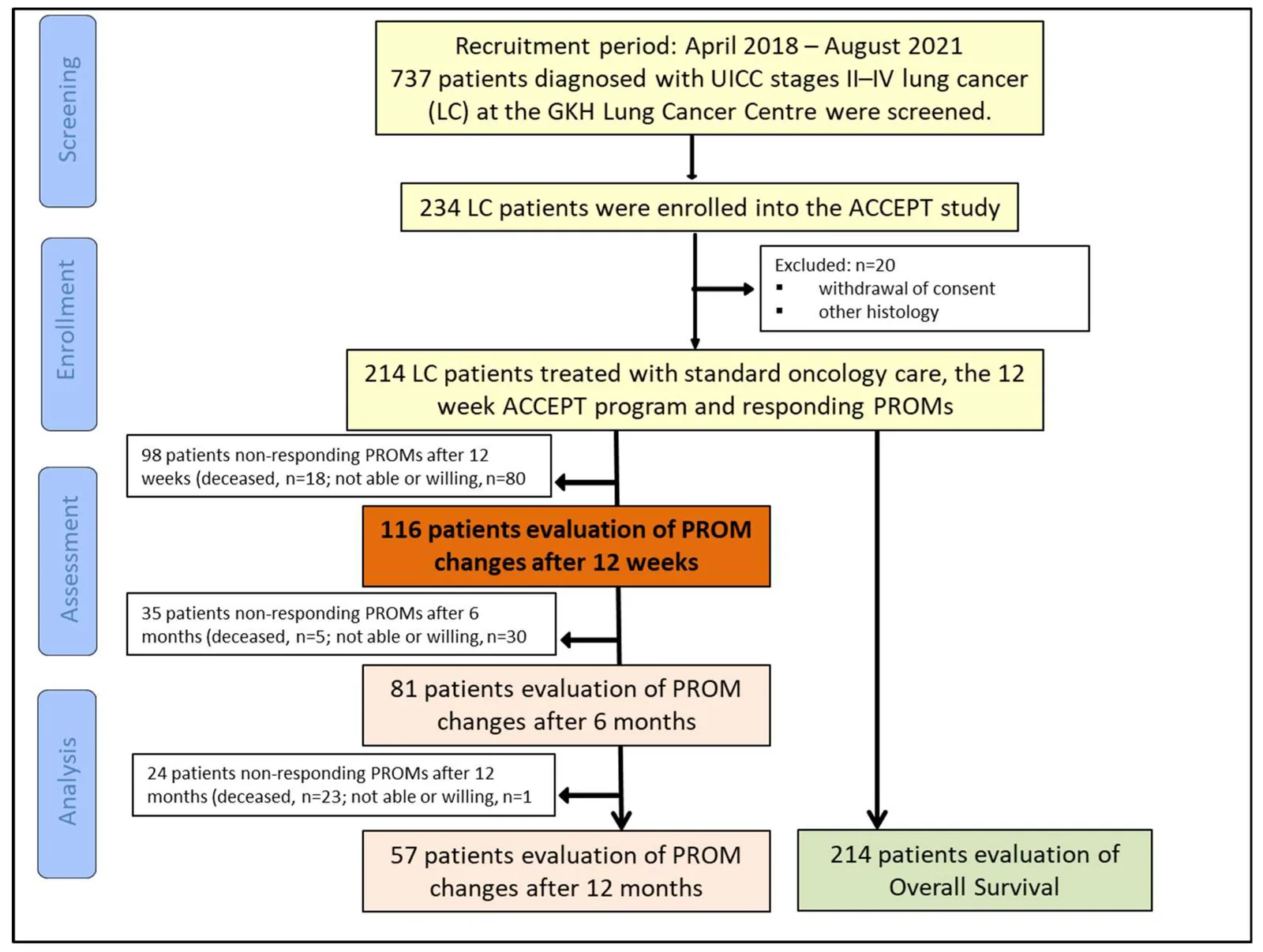
Flowchart of the ACCEPT study. ACCEPT, Additive Integrative Medicine Cancer Concept of Early Supportive or Palliative Lung Cancer Treatment; PROM, Patient-Reported Outcome Measure; UICC, Union for International Cancer Control.

**Figure 3:**
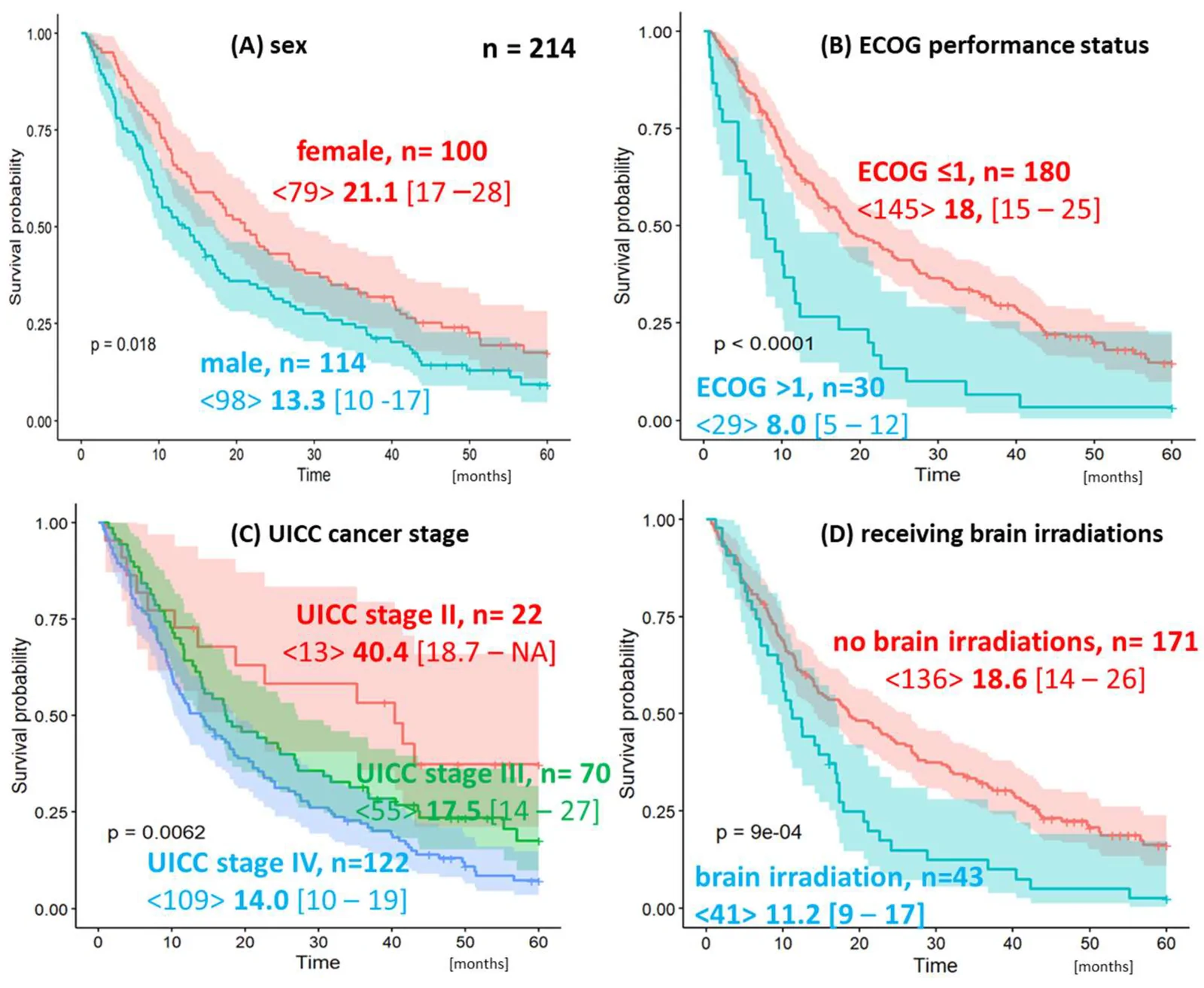
Five-Year Overall Survival. Kaplan–Meier survival curves showing the 5-year survival of LC patients in the entire study cohort, separated by (**A**) sex, (**B**) Eastern Cooperative Oncology Group (ECOG) performance status, (**C**) Union for International Cancer Control (UICC) tumor stage, or (**D**) received brain irradiation. n, numbers, <number of death events>, median survival in months, and [95% CI] are given.

**Figure 4:**
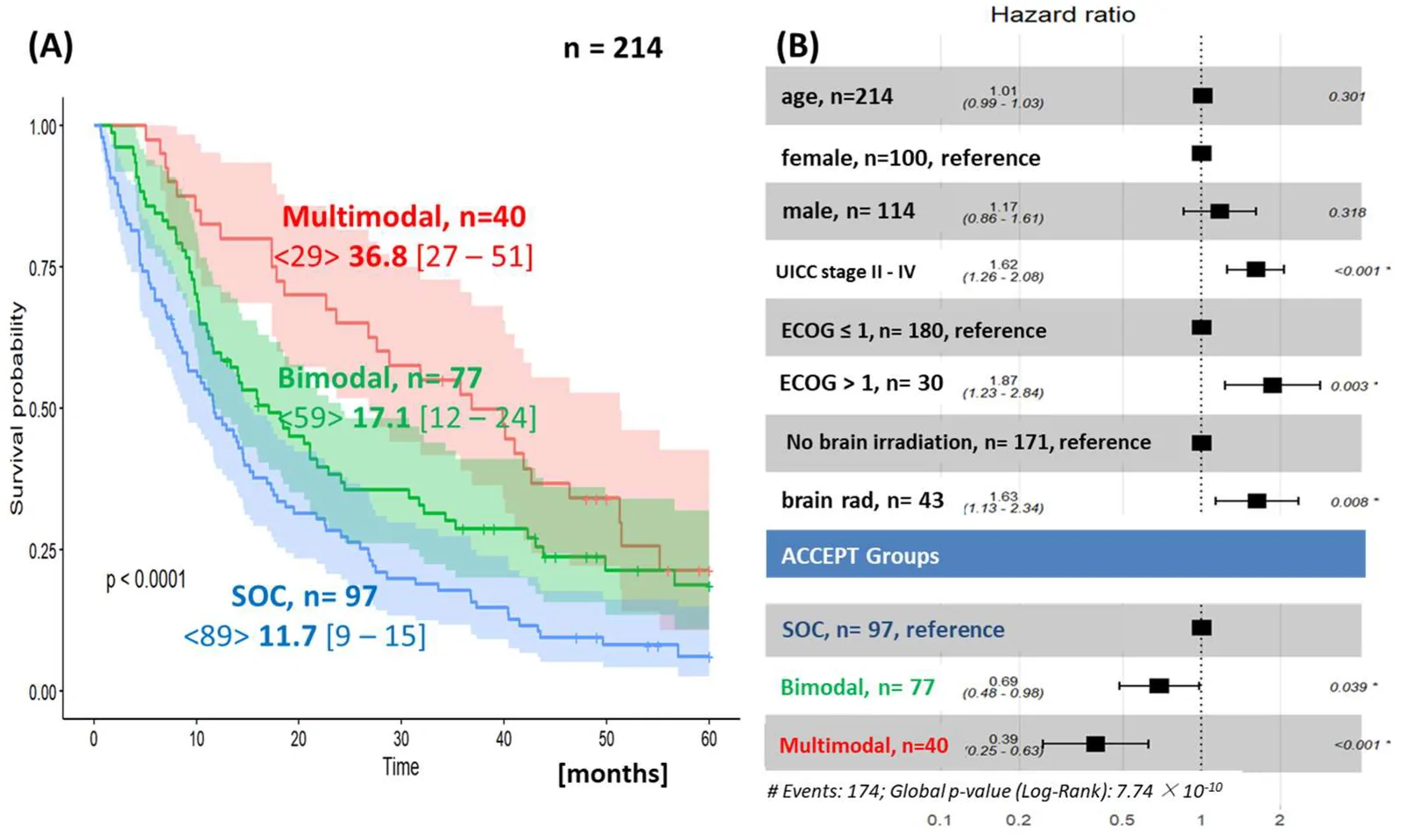
The ACCEPT Program and Overall Survival. (**A**), Kaplan–Meier survival curves showing the 5-year survival of the entire study cohort, separated by participation in multimodal (red), bimodal (green), and standard of care (SOC) (blue). n, numbers, <number of death events>, median survival, and [95% CI] are given. (**B**), Multivariate Cox proportional hazards regression analyses. Numerical variables were age (in years) and UICC tumor stage (II–IV); categorical variables were sex (male, female), ECOG performance stage (ECOG > 2, ECOG ≤ 1), received brain irradiation (no, yes), and ACCEPT Groups (SOC, Bimodal, Multimodal). * Significant *p*-values are indicated. ECOG: Eastern Cooperative Oncology Group performance status; UICC: Union for International Cancer Control.

**Figure 5:**
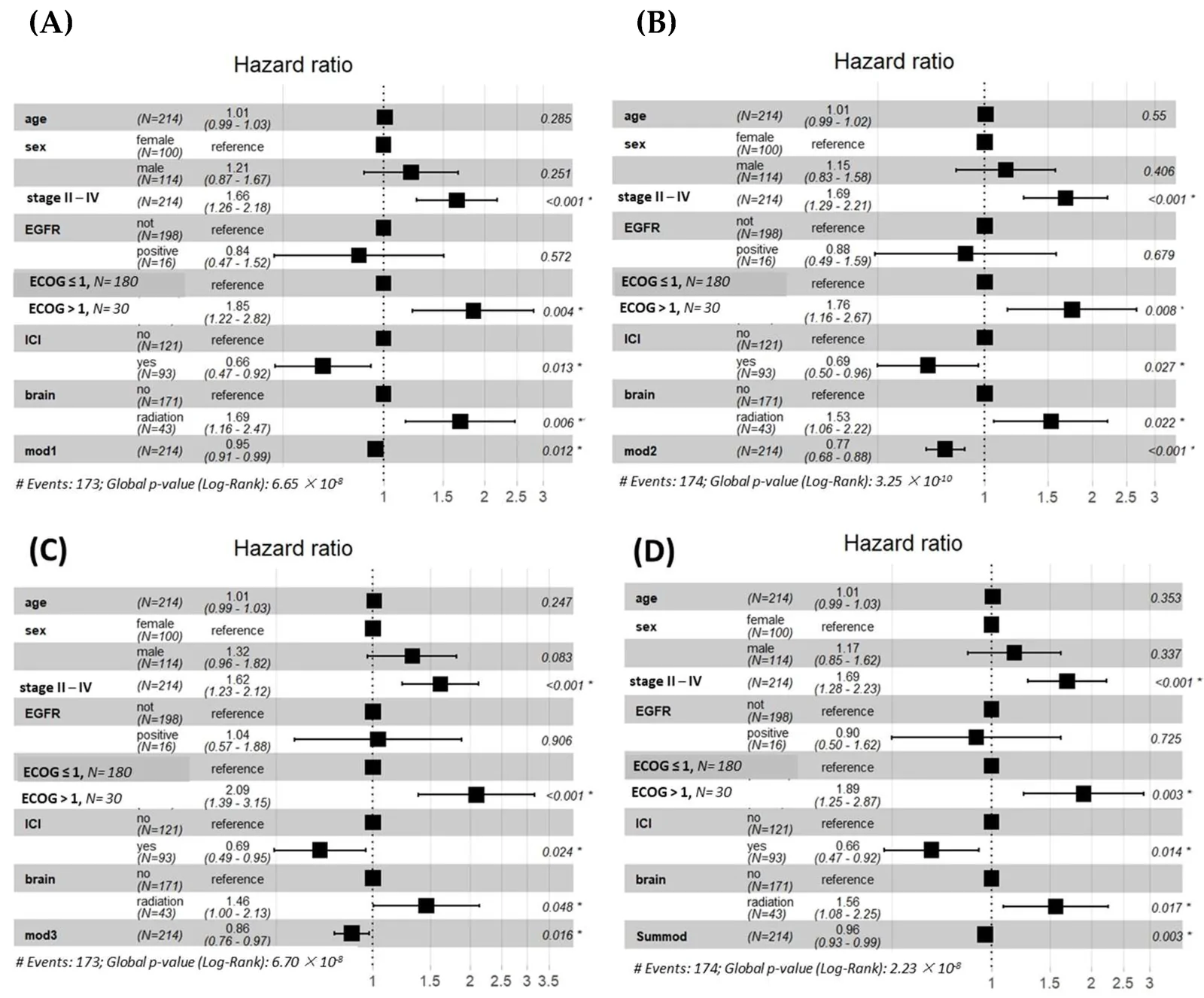
Cox Proportional Hazards Regression Analyses of the ACCEPT Modules on Survival. Adjusted multivariate Cox proportional hazards regression analyses were performed for the entire cohort (n = 214) with respect to the number of ACCEPT module sessions attended. Numerical variables were age (in years), UICC tumor stage (II-IV), and number of attended modules; categorical variables were sex (male, female), EGFR (not positive), ECOG performance stage (ECOG > 2, ECOG ≤ 1), immunotherapy (ICI no, yes), and received brain irradiation (no, yes). (**A**) Module 1, (**B**) Module 2, (**C**) Module 3, and (**D**) the total of Modules 1–3. * Significant *p*-values are indicated: ECOG: Eastern Cooperative Oncology Group performance status; EGFR: epidermal growth factor receptor; ICI: Immune Checkpoint Inhibitor. Summod: total of Modules 1–3.

**Table 1:** Overview of PROMs utilized in the present evaluation.

PROM Tool	Brief Presentation & Domains Covered	Rationale for Selection	Alignment with Study Aims
Lung Cancer-Specific Quality of Life (FACT-L TOI) [17]	A 44-item composite score combining physical well-being, functional well-being, and the lung cancer subscale	Focuses strictly on physical and functional aspects, providing a highly reliable, objective measure of pure clinical and physical efficacy	Core Study Aim: To quantify changes in the physical and functional trajectory of patients undergoing multimodal care
Psychological Distress (HADS) [18]	14-item scale split into two subscales measuring anxiety and depressive symptoms, excluding physical symptoms	Validated for screening psychological distress in physically ill patients without confounding from somatic cancer symptoms	Secondary Aim: To assess the impact of early multimodal supportive care on reducing psychological distress and emotional burden
Sleep Quality (PSQI) [19]	A 19-item self-report instrument assessing sleep quality, latency, duration, efficiency, disturbances, and daytime dysfunction	The global standard for evaluating sleep architecture and sleep disturbances in clinical and oncological populations	Secondary Aim: To monitor sleep quality as a crucial, interdependent component of overall quality of life and symptom distress
Total Fatigue (CFS-D) [20]	A 15-item multi-dimensional scale assessing physical, affective, and cognitive subscales of cancer-related fatigue	Specifically translated and validated for German cancer cohorts to capture the subjective, profound burden of fatigue	Secondary Aim: To evaluate whether early supportive interventions can mitigate the debilitating impact of cancer-related fatigue

Abbreviations: CFS-D, Cancer Fatigue Scale (German version); FACT-L, Functional Assessment of Cancer Therapy-Lung; HADS, Hospital Anxiety and Depression Scale; PROM, Patient-Reported Outcome Measure; PSQI, Pittsburgh Sleep Quality Index; TOI, Trial Outcome Index.

**Table 2:** Characteristics of the ACCEPT cohort.

	Entire Cohort n = 214	SOC n = 97	Bimodal n = 77	Multimodal n = 40
age at inclusion, years			* *p* = 0.012	* *p* = 0.044
median (IQR)	67 (60–74)	71 (63–77)	65 (60–71)	66 (56–71)
sex, n (%)				* *p* = 0.0006
female	100 (47)	35 (36)	37 (48)	28 (70)
male	114 (53)	62 (64)	40 (52)	12 (30)
body mass index, kg/m^2^, n (%)				
normal (<25)	134 (63)	55 (57)	50 (65)	29 (73)
overweight (25–29.9)	61 (29)	32 (33)	23 (30)	6 (15)
obese (≥30)	16 (7)	9 (9)	3 (4)	4 (10)
nd	3 (1)	1 (1)	1 (1)	1 (3)
smoker, n (%)				
current/past	148 (69)	51 (53)	65 (84)	32 (80)
never	15 (7)	4 (4)	4 (5)	7 (18)
nd	51 (24)	42 (43)	8 (10)	1 (3)
education status, n (%)			* *p* = 0.009	* *p* = 0.0006
secondary School	43 (20)	19 (20)	15 (19)	9 (23)
middle School	48 (22)	17 (18)	22 (29)	9 (23)
high School	48 (22)	5 (5)	23 (30)	20 (50)
nd	75 (35)	56 (58)	17 (22)	2 (5)
UICC tumor stage, n (%)				* *p* = 0.013
UICC stage II	22 (10)	13 (13)	8 (10)	1 (3)
UICC stage III	70 (33)	35 (36)	27 (35)	8 (20)
UICC stage IV	122 (57)	49 (51)	42 (55)	31 (78)
ECOG performance status, n (%)			* *p* = 0.012	* *p* = 0.022
ECOG ≤ 1	180 (84)	72 (74)	70 (91)	38 (95)
ECOG > 1	30 (14)	22 (23)	6 (8)	2 (5)
nd	4 (2)	3 (3)	1 (1)	0
Preexisting Comorbidities, n (%)			* *p* = 0.0002	* *p* = 0.0006
COPD	79 (37)	52 (54)	19 (25)	8 (20)
Cardiovascular diseases	13 (6)	8 (8)	3 (4)	2 (5)

ECOG, Eastern Cooperative Oncology Group; IQR, interquartile range; n, number; UICC, Union for International Cancer Control; nd, not determined. * Significant *p*-values from Pearson chi-square tests relating to the SOC group are indicated.

**Table 3:** Comparison of PROMs between responding and non-responding cohorts.

	PROMs Responding Cohort n = 116	Non-Responding Cohort n = 98	*p*-Value
age at inclusion, years, median (IQR)	67 (59–73)	68 (62–74)	0.487
sex, n (%)			
female	65 (56)	35 (36)	0.0046 *
male	51 (44)	63 (64)	
smoker, n (%)			
Current/past	101 (87)	47 (48)	
never	8 (7)	7 (7)	0.378
nd	7 (6)	44 (45)	
Body mass index, kg/m^2^, n (%)			
normal (<25)	78 (67)	56 (57)	
overweight (25–29.9)	29 (25)	32 (33)	0.356
obese (≥30)	8 (7)	8 (8)	
nd	1 (1)	2 (2)	
Education Status, n (%)			
Secondary School	31 (27)	12 (12)	
Middle School	38 (33)	10 (10)	0.194
High School	30 (26)	18 (18)	
nd	17 (15)	58 (59)	
UICC tumor stage, n (%)			
UICC stage II	10 (9)	12 (12)	
UICC stage III	39 (34)	31 (32)	0.681
UICC stage IV	67 (58)	55 (56)	
nd	0	0	
ECOG performance status, n (%)			
0	63 (54)	39 (40)	
0.5–1	38 (33)	40 (41)	0.057
1.5–2	12 (10)	18 (18)	
nd	3 (3)	1 (1)	
Baseline PROMs, mean (SD)			
Physical Function and Well-Being/ FACT-L TOI (0–84)	52.6 (15.0)	51.3 (16.1)	0.600
Psychological Distress (Total) HADS-Total (0–42)	13.7 (8.0)	13.2 (7.2)	0.690
Sleep Quality (Total) /PSQI-Total (0–21)	6.3 (3.0)	7.0 (3.6)	0.181
Total Fatigue/ CFS-D-Total (0–60)	24.4 (11.4)	24.7 (10.3)	0.884

ECOG, Eastern Cooperative Oncology Group; IQR, interquartile range; n, number; UICC, Union for International Cancer Control; nd, not determined. * Significant *p*-values from Pearson chi-square tests between responding and non-responding cohorts are indicated.

**Table 4:** Histology and treatments of the entire ACCEPT cohort and groups.

n = 214	Entire Cohort n = 214	SOC n = 97	Bimodal n = 77	Multimodal n = 40
Histology, n (%)				
Non-small cell lung cancer (NSCLC)	177 (83)	80 (83)	64 (83)	33 (83)
Small cell lung cancer (SCLC)	35 (16)	16 (16)	12 (16)	7 (17)
Other ^#^	2 (1)	1 (0.5)	1 (1)	0
				* *p* = 0.001
Molecular pathology, Yes, n (%)	93 (43)	32 (33)	37 (48)	24 (60)
*KRAS* positive, Yes	26 (12)	8 (8)	13 (17)	5 (13)
				* *p* = 0.0009
*EGFR* positive, Yes	16 (7)	3 (3)	4 (5)	9 (23)
*BRAF* positive, Yes	2 (1)	0	0	2 (5)
*ALK* positive, Yes	2 (1)	0	1 (1)	1
Primary oncological treatment goal, n (%)				* *p* = 0.033
palliativ	126 (59)	52 (54)	44 (57)	30 (75)
curativ	88 (41)	45 (46)	33 (43)	10 (25)
Received treatment, n (%)				
Chemotherapy	161 (75)	68 (70)	54 (70)	29 (73)
Immunotherapy	93 (43)	38 (39)	38 (49)	17 (43)
				* *p* < 0.0001
Targeted	21 (10)	3 (3)	6 (8)	12 (30)
Brain irradiation				
Yes, n (%)	43 (20)	17 (18)	16 (21)	10 (25)
12-week ACCEPT program, number of sessions attended				
Education Module 1: Completed sessions (max. 12), mean (SD)	2.7 (4.1)	0	3.1 (3.6)	8.4 (4.4)
Coping Module 2: Completed sessions (max. 3), mean (SD)	1.4 (1.4)	0	2.4 (0.8)	3.0 (0.4)
Arts Module 3: Completed sessions (max. 4), mean (SD)	0.6 (1.4)	0	0.1 (0.3)	3.3 (1.7)
Total for Modules 1–3, mean (SD)	4.7 (6.1)	0	5.5 (3.8)	13.9 (5.6)

^#^ other histology: 1 combined carcinoma; 1 sarcoid carcinoma; nd: not determined. * Significant *p*-values from Pearson chi-square tests relating to the SOC group are indicated.

**Table 5:** PROMs of the ACCEPT Study Cohort.

ACCEPT Study Cohort n = 116	SOC n = 43	Bimodal n = 38	Multimodal n = 35
12-Week ACCEPT Program, Number of Sessions Attended			
Education Module 1: Completed sessions (max. 12), mean (SD)	0	3.2 (3.1)	8.8 (4.1)
Coping Module 2: Completed sessions (max. 3), mean (SD)	0	2.9 (0.4)	3.0 (0.3)
Arts Module 3: Completed sessions (max. 4), mean (SD)	0	0.1 (0.5)	3.2 (1.6)
Baseline PROMs, mean (SD)	SOC	Bimodal	Multimodal
Physical Function and Well-Being/FACT-L TOI (0–84)	54.61 (16.08)	50.43 (13.25)	52.24 (14.77)
Lung Cancer-Specific Quality of Life/FACT-Lung Total (0–136)	93.68 (22.70)	87.34 (17.14)	89.18 (21.13)
General Quality of Life/ FACT-Global Total (0–108)	75.32 (19.67)	68.11 (15.21)	70.12 (17.55)
Psychological Distress (Total)/ HADS-Total (0–42)	11.39 (6.95)	15.92 (8.01)	14.09 (8.34)
Anxiety/HADS-Anxiety (0–21)	5.81 (4.09)	8.64 (4.41)	7.69 (4.62)
Depression/HADS-Depression (0–21)	5.58 (3.24)	7.29 (4.13)	6.40 (3.97)
Sleep Quality/PSQI-Total (0–21)	5.73 (3.04)	7.00 (3.03)	6.37 (2.88)
Total Fatigue/CFS-D-Total Fatigue (0–60)	23.43 (12.12)	25.95 (10.02)	24.07 (11.59)
Physical Fatigue (0–24)	11.17 (5.81)	12.76 (4.82)	11.83 (5.78)
Cognitive Fatigue (0–20)	5.67 (3.78)	6.11 (3.85)	6.54 (4.16)
Affective Fatigue (0–16)	6.60 (3.74)	7.06 (3.05)	5.71 (2.75)
^#^ PROM changes, mean (SD)	SOC	Bimodal	Multimodal
ΔPhysical Function and Well-Being (ΔTOI)	−1.43 (14.61)	4.97 * (12.18)	12.91 *** (12.44)
ΔLung Cancer-Specific Quality of Life (ΔFACT-Lung Total)	−4.85 (26.04)	8.09 * (16.43)	16.91 *** (16.27)
ΔGeneral Quality of Life (ΔFACT-Global Total)	−2.44 (17.65)	6.59 * (13.70)	13.85 *** (13.58)
ΔPsychological Distress (ΔHADS-Total)	0.75 (6.79)	−4.01 ** (6.27)	−6.26 *** (5.75)
ΔAnxiety (ΔHADS-Anxiety)	0.64 (4.14)	−2.23 ** (3.55)	−3.59 *** (3.37)
ΔDepression (ΔHADS-Depression)	0.12 (3.14)	−1.77 ** (3.53)	−2.68 *** (2.79)
ΔSleep Quality (ΔPSQI-Total)	0.44 (2.05)	−1.72 ** (3.33)	−2.26 *** (2.74)
ΔTotal Fatigue (ΔCFS-D-Total Fatigue)	0.79 (8.12)	−1.09 (9.03)	−4.78 ** (8.31)
ΔPhysical Fatigue	0.43 (4.53)	−0.59 (4.10)	−2.31 ** (4.43)
ΔCognitive Fatigue	0.27 (2.82)	−0.10 (3.02)	−0.51 (2.95)
ΔAffective Fatigue	−0.26 (3.30)	−0.53 (3.48)	−1.97 ** (2.61)

ACCEPT, Additive Integrative Medicine Cancer Concept of Early Supportive or Palliative Lung Cancer Treatment; ARTs, art therapy; SOC, standard of care; SD, standard deviation. Significant *p*-values (Student’s *t*-tests, two-sided, paired) are indicated: ***: *p*-value < 0.0001; **: *p*-value < 0.005; *: *p*-value < 0.05. ^#^ Positive PROM changes indicate improvement for FACT-TOI and worsening for symptom scales (HADS, PSQI, CFS-D); negative changes indicate the opposite. Significant improvements are highlighted in bold. CFS-D, Cancer fatigue scale, German version; FACT-L, Functional Assessment of Cancer Therapy-Lung; HADS, Hospital Anxiety and Depression Scale PROM, patient-reported outcome measure; PSQI, Pittsburgh Sleep Quality Index.

**Table 6:** Propensity scores for PROM changes at 12 weeks.

Outcome	Comparison	Estimate (β)	95%CI	*p*-Value
Δphysical function and well-being (ΔTOI)	Bimodal vs. SOC	+3.83	[−3.37, +11.03]	0.298
	Multimodal vs. SOC	+11.52	[+4.29, +18.75]	0.002 *
Δpsychological distress (ΔHADS)	Bimodal vs. SOC	−3.86	[−6.97, −0.74]	0.016 *
	Multimodal vs. SOC	−6.18	[−9.41, −2.96]	<0.001 *

Estimates are based on inverse probability of treatment weighting (IPTW) using multinomial propensity scores. Models were adjusted for baseline PROMs, sex, and ECOG performance status. The SOC group served as the reference category. CI = confidence interval. * Significant *p*-values are indicated.

**Table 7:** PROM changes at 6 and 12 months.

**ACCEPT Study Cohort** **6 Months, n = 81**	**SOC** **n = 28**	**Bimodal** **n = 25**	**Multimodal** **n = 28**
ΔTOI, mean (SD)	−4.07 (15.78)	4.86 (13.94)	9.26 * (15.13)
ΔHADS-Total, mean (SD)	1.77 (5.31)	−2.14 (5.98)	−5.19 ** (4.80)
ΔHADS-Anxiety, mean (SD)	1.08 (3.36)	−1.29 (3.51)	−3.04 ** (2.92)
ΔHADS-Depression, mean (SD)	0.63 (2.91)	−0.85(3.01)	−2.15 ** (2.73)
ΔPSQI-Total, mean (SD)	0.85 (3.30)	−1.23 (3.82)	−1.82 * (3.15)
ΔCFS-D-total fatigue, mean (SD)	1.34 (10.43)	−0.54 (7.77)	−1.31 (10.15)
**ACCEPT Study Cohort** **12 Months, n = 57**	**SOC** **n = 15**	**Bimodal** **n = 18**	**Multimodal** **n = 24**
ΔTOI, mean (SD)	−5.07 (14.01)	4.00 (8.29)	8.17 * (17.79)
ΔHADS-Total, mean (SD)	1.14 (4.70)	−4.36 * (5.48)	−3.82 * (5.90)
ΔHADS-Anxiety, mean (SD)	1.00 (2.42)	−3.04 * (3.40)	**−1.79 (4.27)**
ΔHADS-Depression, mean (SD)	0.14 (3.09)	−1.33 (2.64)	−2.03 ** (2.38)
ΔPSQI-Total, mean (SD)	0.00 (2.50)	−2.06 * (3.51)	−1.33 * (2.48)
ΔCFS-D-total fatigue, mean (SD)	2.50 (14.39)	−1.83 (8.20)	−1.12 (10.11)

ACCEPT, Additive Integrative Medicine Cancer Concept of Early Supportive or Palliative Lung Cancer Treatment; SOC, standard of care; SD, standard deviation. Significant *p*-values (Student’s *t*-tests, two-sided, paired) are indicated: **: *p*-value < 0.005; *: *p*-value < 0.05. Significant improvements are highlighted in bold. CFS-D, Cancer fatigue scale, German version; HADS, Hospital Anxiety and Depression Scale PROM, patient-reported outcome measure; PSQI, Pittsburgh Sleep Quality Index.

**Table 8:** Association factors for PROM changes at 12 weeks.

**ACCEPT Cohort n = 116**		**ΔHADS** **(0–42)**	**ΔFACT-L Total** **(0–136)**	**ΔTOI** **(0–84)**
Sex: female; male reference		−1.089	6.530	5.013 *
Age, in years		−0.093	0.196	0.093
ECOG > 1, ECOG ≤ 1 reference		1.751	−6.873	−7.107 ^+^
Baseline PROMs		−0.451 ***	−0.381 ***	−0.419 ***
Primary oncological treatment goal curative, palliative reference		1.416	−0.784	−3.115
		Health Education Module 1 (max. 12)	Psychooncology Module 2 (max. 3)	ARTs Module 3 (max. 4)
Number of module sessions attended		−0.315 *	4.607 **	2.582 ***
Adjusted R^2^		**0.320**	**0.261**	**0.314**
Adjusted multivariate regression analyses Association factors per number of module sessions				
**ACCEPT-program** **12 weeks**	**Health Education** **Module 1**	**Psychooncology** **Module 2**	**ARTs** **Module 3**	**Total for** **Modules 1–3**
ΔTOI	0.540 ^+^	2.418 *	2.582 ***	0.566 **
ΔFACT-Lung Total	0.791 ^+^	4.607 **	3.845 **	0.899 **
ΔFACT-Global Total	0.482	2.874 **	2.886 ***	0.603 **
ΔHADS-Total	−0.315 *	−1.528 ***	−1.243 ***	−0.296 **
ΔHADS-Anxiety	−0.174 *	−0.859 **	−0.775 ***	−0.179 **
ΔHADS-Depression	−0.139 *	−0.637 **	−0.479 **	−0.116 **
ΔPSQI-Total	−0.081	−0.384 ^+^	−0.350 *	−0.083 *
ΔCFS-D-Total Fatigue	−0.277	−0.729	−1.368 **	−0.250 *
ΔPhysical Fatigue	−0.098	−0.330	−0.570 *	−0.102
ΔCognitive Fatigue	−0.037	−0.083	−0.289 ^+^	−0.035
ΔAffective Fatigue	−0.119 ^+^	−0.173	−0.514 **	−0.097 *

Multivariate linear regression analyses, adjusted for age, sex, the respective baseline PROMs, ECOG, and intended curative versus palliative treatment, were performed for participation in the number of sessions in the three modules for PROM changes at 12 weeks. Significant *p*-values are indicated: ***: *p*-value < 0.001; **: *p*-value < 0.01; *: *p*-value < 0.05; ^+^: *p*-value < 0.1. Significant improvements are highlighted in bold. ECOG, Eastern Cooperative Oncology Group; FACT-L, Functional Assessment of Cancer Therapy-Lung; HADS, Hospital Anxiety and Depression Scale; PROM, patient-reported outcome measure; PSQI, Pittsburgh Sleep Quality Index; TOI, Trial Outcome Index.

**Table 9:** Association factors for PROM changes at 6 and 12 months.

Adjusted Multivariate Regression Analyses Association Factors per Number of Module Sessions				
**ACCEPT-Program** **6 Months Follow-up** **n = 81**	**Health Education** **Module 1**	**Psychooncology** **Module 2**	**ARTs** **Module 3**	**Total for** **Modules 1–3**
ΔTOI	0.711 ^+^	2.107	2.079 *	0.504 *
ΔHADS-Total	−0.274 ^+^	−1.161 *	−1.048 **	−0.230 *
ΔHADS-Anxiety	−0.166 ^+^	−0.516 ^+^	−0.667 **	−0.138 *
ΔHADS-Depression	−0.115	−0.603 *	−0.395 *	−0.093 ^+^
ΔPSQI-Total	−0.213 *	−0.484 ^+^	−0.604 **	−0.165 **

**ACCEPT-program** **12 months follow-up** **n = 57**	**Health Education** **Module 1**	**Psychooncology** **Module 2**	**ARTs** **Module 3**	**Total for** **Modules 1–3**
ΔTOI	0.478	3.424 *	−0.081	0.508 ^+^
ΔHADS-Total	0.079	−0.881	−0.393	−0.056
ΔHADS-Anxiety	0.085	−0.370	−0.075	0.002
ΔHADS-Depression	−0.003	−0.491	−0.305	−0.054
ΔPSQI-Total	0.014	−0.365	−0.297	−0.083

Multivariate linear regression analyses, adjusted for age, sex, the respective baseline PROMs, ECOG, and intended curative versus palliative treatment, were performed for participation in the number of sessions in the three modules for PROM changes at 6 or 12 months. Significant *p*-values are indicated: **: *p*-value < 0.01; *: *p*-value < 0.05; ^+^: *p*-value < 0.1. Significant improvements are highlighted in bold. ACCEPT, Additive Integrative Medicine Cancer Concept of Early Supportive or Palliative Lung Cancer Treatment; ARTs, art therapy; ECOG, Eastern Cooperative Oncology Group; HADS, Hospital Anxiety and Depression Scale; PROM, patient-reported outcome measure; PSQI, Pittsburgh Sleep Quality Index; TOI, Trial Outcome Index.

## Data Availability

The datasets that support the findings in this article are not publicly available for privacy and security reasons but can be obtained from the corresponding authors upon reasonable request. All relevant data are included in this manuscript.
